# Sex bias in CNS autoimmune disease mediated by androgen control of autoimmune regulator

**DOI:** 10.1038/ncomms11350

**Published:** 2016-04-13

**Authors:** Meng-Lei Zhu, Pearl Bakhru, Bridget Conley, Jennifer S. Nelson, Meghan Free, Aaron Martin, Joshua Starmer, Elizabeth M. Wilson, Maureen A. Su

**Affiliations:** 1 Division of Endocrinology, Department of Pediatrics, School of Medicine, University of North Carolina at Chapel Hill, Chapel Hill, North Carolina 27599, USA; 2 Department of Microbiology and Immunology, School of Medicine, University of North Carolina at Chapel Hill, Chapel Hill, North Carolina 27599, USA; 3 Lineberger Comprehensive Cancer Center, University of North Carolina at Chapel Hill, Chapel Hill, North Carolina 27599, USA; 4 Division of Cardiothoracic Surgery, Department of Surgery, School of Medicine, University of North Carolina at Chapel Hill, Chapel Hill, North Carolina 27599, USA; 5 Department of Medicine, School of Medicine, University of North Carolina at Chapel Hill, Chapel Hill, North Carolina 27599, USA; 6 Department of Genetics, University of North Carolina at Chapel Hill, Chapel Hill, North Carolina 27599, USA; 7 Department of Biochemistry and Biophysics, School of Medicine, University of North Carolina at Chapel Hill, Chapel Hill, North Carolina 27599, USA

## Abstract

Male gender is protective against multiple sclerosis and other T-cell-mediated autoimmune diseases. This protection may be due, in part, to higher androgen levels in males. Androgen binds to the androgen receptor (AR) to regulate gene expression, but how androgen protects against autoimmunity is not well understood. Autoimmune regulator (Aire) prevents autoimmunity by promoting self-antigen expression in medullary thymic epithelial cells, such that developing T cells that recognize these self-antigens within the thymus undergo clonal deletion. Here we show that androgen upregulates Aire-mediated thymic tolerance to protect against autoimmunity. Androgen recruits AR to Aire promoter regions, with consequent enhancement of Aire transcription. In mice and humans, thymic Aire expression is higher in males compared with females. Androgen administration and male gender protect against autoimmunity in a multiple sclerosis mouse model in an Aire-dependent manner. Thus, androgen control of an intrathymic Aire-mediated tolerance mechanism contributes to gender differences in autoimmunity.

## Abstract (web summary)

Males are less susceptible to autoimmune diseases due to immunomodulatory effects of androgen. Here the authors show that androgen receptor upregulates Aire and Aire-dependent transcription in the thymic epithelium, and that Aire is required for androgen-mediated suppression of experimental autoimmune encephalitis.

## Graphical abstract

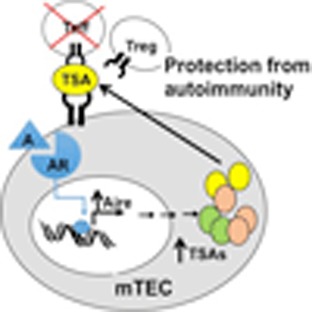

Males are at lower risk for many autoimmune diseases compared with females. In multiple sclerosis, for example, the sex ratio of females to males exceeds 3:1 (ref. 1). This gender imbalance may reflect higher androgen levels in males, since androgen protects against autoimmunity in mice and humans (refs 2, 3, 4); reviewed in ref. 5). Biologically active androgens can exert their effects by binding to the androgen receptor (AR) to regulate target gene transcription. The identity of androgen/AR-regulated immune genes that confer autoimmune protection, however, is not known.

In the thymus, the autoimmune regulator (Aire) gene enforces T-cell self-tolerance in part by promoting expression of tissue-specific antigens (TSAs) in medullary thymic epithelial cells (mTECs; ref. 6; reviewed in ref. 7). Developing thymocytes that recognize these TSAs with high affinity undergo negative selection, thus preventing the release of self-reactive T cells into the periphery. Aire may also prevent autoimmunity through the additional mechanisms, such as promoting regulatory T-cell development^8^ and expression of chemokines important in mediating T-cell negative selection^9^. The critical role for Aire in preventing autoimmunity is highlighted by the development of spontaneous, multi-organ autoimmune disease in Aire-deficient humans and mice. Homozygous Aire mutations in humans result in autoimmune polyendocrinopathy syndrome type 1 (or autoimmune polyendocrinopathy candidiasis ectodermal dysplasia), a disease that predisposes to a constellation of T-cell-mediated autoimmune manifestations^10^. In parallel, homozygous Aire mutations in mice result in increased frequency of self-reactive T cells in the periphery and T-cell-mediated destruction of multiple organs. Thus, Aire is integral in maintaining T-cell tolerance.

While complete loss of Aire function results in autoimmune disease, quantitative decreases in Aire also predispose to autoimmunity. Several lines of evidence suggest that Aire regulation of T-cell tolerance is dose dependent and non-binary. First, mutations that quantitatively decrease Aire expression^11^ or function^12^ predispose to autoimmunity. Second, Aire expression levels are highly variable between individuals in the general population and strongly correlate with the TSA expression levels^13^. Quantitative decreases in Aire-mediated TSA expression in the thymus, furthermore, predict the development of autoimmunity^14^,^15^. Third, Aire polymorphisms in humans that incrementally decrease Aire expression are associated with increased development of autoimmune disease^16^. Together, these findings suggest that factors that quantitatively regulate Aire expression may determine autoimmunity predisposition.

On the basis of these findings, we hypothesized that androgen/AR complexes may upregulate Aire expression to protect against autoimmune disease. In support of this hypothesis, we show that male mice and humans express increased Aire in the thymus. Aire expression in male mice was reduced with castration and genetic AR deficiency, suggesting a role for testicular androgen and AR in upregulating Aire in males. Consistent with this, androgen administration increased thymic Aire expression *in vitro* and *in vivo*. Finally, we show that androgen administration and male gender protected against autoimmunity in a mouse model of multiple sclerosis through an Aire-dependent mechanism. Together, these results suggest that androgen regulation of the intrathymic Aire tolerance mechanisms alters predisposition to autoimmunity.

## Results

### Males express increased Aire in human and mouse thymus

**Figure 1:**
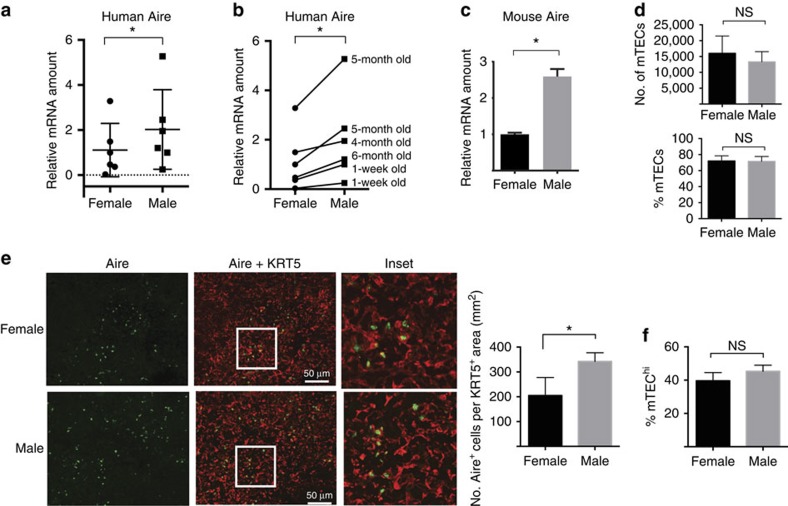
Male thymus expresses increased Aire in humans and mice. (**a**) Relative Aire mRNA expression in human thymus of male and female infants (<6 months of age). Relative expression was measured by quantitative RT–PCR relative to cytokeratin 5, a thymic medulla marker. *n*=6 for each group. Error bars represent s.d. (**b**) Relative Aire mRNA expression in **a** displayed as age-matched male–female pairs. Ages are indicated at the right of each pair. (**c**) Relative mRNA expression of Aire in sorted mTECs from male versus female thymi of 6-week-old mice. A total of 4–10 mice were pooled per sample. Data shown are representative of four independent experiments. Error bars represent s.d. (**d**) Top: absolute numbers of total mTECs (CD45^−^ MHCII^+^, Ly51^low^) by flow cytometry in 8-week-old male versus female mice. Bottom: frequency of total mTECs within CD45^−^ MHCII^+^ stromal cells. Thymi from pools of 4 mice per group were used in each replicate, with three replicates total. (**e**) Left: representative immunofluorescent images of 8-week-old male and female thymus sections stained with anti-Aire (green) and anti-cytokeratin 5 (K5) antibodies. Right: quantification of Aire+ cells per K5+ area for females and males. *n*=4 mice per group. Error bars represent s.e.m. (**f**) Ratio of mTEC^hi^ (CD45^−^ MHCII^high^, Ly51^low^) to mTEC^lo^ (CD45^−^ MHCII^low^, Ly51^low^) in males versus females. *n*=3 for each group. Error bars represent s.e.m. For all data sets, unpaired Student’s *t*-test was used for comparisons except in **b**, in which paired Student’s *t*-test was used. *P<0.05. NS, not significant.

Males are protected from the development of multiple autoimmune diseases compared with females^17^. Since Aire plays a critical role in protecting from autoimmunity, we hypothesized that increased androgen in males may upregulate Aire expression to contribute to this protection. To test this, we first compared thymic Aire expression in male versus female subjects, using human thymus tissue removed from infants (<6 months of age) during the course of cardiac surgery. Human infants in the first 6 months of life undergo ‘mini-puberty’, with high circulating androgen levels in male infants^18^,^19^. Thus, thymus tissue in male infants during this period is exposed to high androgen levels. Relative Aire messenger RNA (mRNA) expression was determine by quantitative PCR with reverse transcription (RT–PCR) and normalized to cytokeratin 5 (KRT) 5, a housekeeping gene expressed by thymic medulla. On average, male thymus expressed higher levels of Aire mRNA compared with female thymus (Fig. 1a). When human subjects were paired by age, Aire expression was consistently higher in the male thymus samples (Fig. 1b). In parallel, we flow-sorted mTECs from male and female thymus of 6-week-old adult mice and compared Aire mRNA expression. mTECs from male mice expressed higher mRNA levels of Aire (Fig. 1c). These differences were not due to gross alterations in thymic epithelial numbers, since absolute numbers and frequency of mTECs by flow cytometry were unchanged between males and females (Fig. 1d). To compare Aire protein expression, we performed immunofluorescent staining of thymic sections from male and female mice. Male thymic sections showed a modest but significant increase in Aire-expressing (Aire^+^) cells per KRT5^+^ thymic medullary area (Fig. 1e). Because Aire is expressed largely in differentiated mTECs that express high levels of MHCII (mTEC^hi^; (ref. 20)), this increase could be either due to an increased proportion of mTECs that are mature (and therefore express Aire) or to an increased Aire expression per mature mTEC. To distinguish between these two possibilities, we compared the frequency of mTEC^hi^ cells by flow cytometry. No differences in mTEC^hi^ frequencies were noted between males and females (Fig. 1f). Together, these findings suggest increased Aire expression per mTEC at the mRNA and protein level.

**Figure 2:**
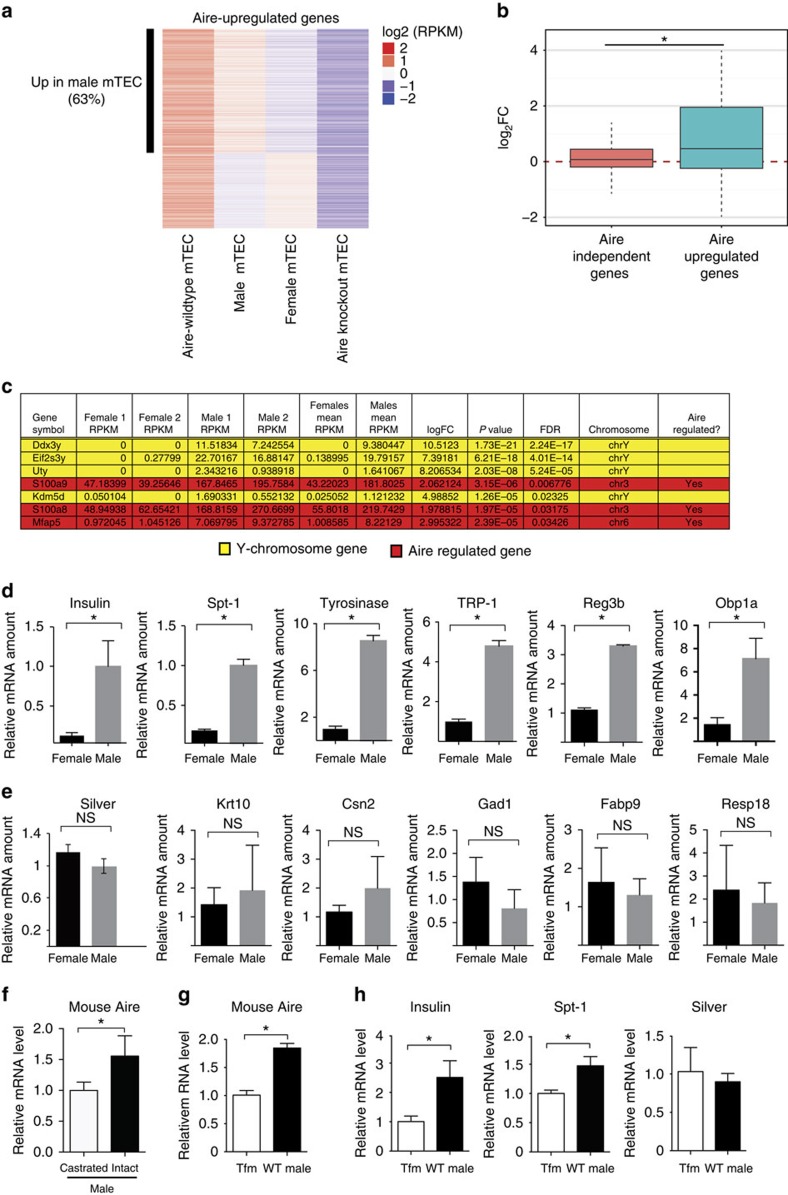
Male thymus expresses increased Aire-regulated TSAs in mice. (**a**) Heatmap of Aire-upregulated genes. Extreme left and right columns: expression of genes (log_2_RPKM) shown to be upregulated in Aire wild-type (WT, left) compared with Aire knockout mTECs (right) in ref. 21. Middle two columns: expression of Aire-upregulated genes in mTECs of males (left) compared with females (right). Left bar demarcates Aire-upregulated genes that are increased in male mTECs (63%). (**b**) Box plot of average fold change (male over female) for expression of Aire-independent versus Aire-dependent genes in which message was detected. *P=3.17e−15. (**c**) List of top seven genes upregulated in males (FDR <0.05). RNA for sequencing was obtained from flow-sorted mTECs from male and female mice. Ten mice were pooled per replicate with two replicates per group. (**d**,**e**) Relative mRNA expression of TSAs in sorted mouse mTECs from male versus female thymic stroma. Six Aire-dependent TSAs ((**d**) insulin, Spt-1, tyrosinase, TRP-1, Reg3b and Obp1a) and six Aire-independent TSAs ((**e**) Silver, Krt10, Csn2, Gad1, Fabp9 and Resp18) were tested. A total of 4–10 mice were pooled per sample. Data shown are representative of two independent experiments. (**f**) Relative Aire mRNA expression in thymic stromal cells from castrated versus unmanipulated (intact) male mice measured by quantitative RT–PCR. (**g**,**h**) Relative Aire (**g**) and TSA (**h**) (insulin, Spt-1 and Silver) mRNA expression in isolated thymic stroma from AR-deficient testicular feminized AR^Tfm^/Y (Tfm) mice or WT male littermate controls measured by quantitative RT–PCR. For (**f**–**h**), three mice were pooled for each sample, and representative of two independent experiments are shown. Thymic stromal cells were used as starting material. For (**d**–**h**), unpaired Student’s *t*-test was used for comparisons. For (**d**–**h**), error bars represent s.d. *P<0.05. FDR, false discovery rate; NS, not significant.

In addition, mTECs from male mice expressed higher mRNA levels of Aire-regulated TSAs. We utilized RNA sequencing to obtain a global representation of the mTEC transcriptome in 6–8-week-old male versus female mice. Among the 2,760 Aire-upregulated transcripts with detectable transcripts in our samples^21^, 1,721 (63%) were higher in mTECs from males compared with females (Fig. 2a). In addition, log_2_fold change of male over female expression levels was higher for Aire-upregulated genes^21^ compared with Aire-independent genes (*P*=3.17e−15; unpaired Student’s *t*-test; Fig. 2b), which suggests that male gender has a generally positive impact on the Aire-regulated transcripts. Of the top, seven genes induced in males (false discovery rate <0.05), four genes were located on the Y chromosome, which provided internal validation for this comparison between the genders. Of the remaining genes, all three have been reported to be Aire regulated^21^,^22^ (Fig. 2c). We next utilized quantitative RT–PCR to compare expression levels of specific Aire-regulated TSAs. Quantitative RT–PCR analysis of the Aire-regulated TSAs insulin, tyrosinase, TRP-1, Spt-1, Reg3b and OBP1a showed higher TSA expression in male mTECs^6^,^23^,^24^ (Fig. 2d), whereas expression of the Aire-independent TSAs Silver, Krt10, Csn2, Gad1, FABP9 and Resp18 (refs 23, 24, 25) was similar between male and female mTECs (Fig. 2e).

Multiple factors may contribute to the gender difference in Aire expression, including chromosomal, environmental and hormonal factors^5^. We chose to focus on the potential effects of androgen on Aire expression because of the strong existing evidence that androgen is protective against autoimmunity^5^. To test the possibility that testicular androgen may contribute to increased male Aire expression, we castrated 2-week-old C57BL/6 wild-type (WT) male mice and measured Aire expression levels 2 weeks later. Castration decreased Aire mRNA expression in thymic stromal cells compared with intact, age-matched males (Fig. 2f), suggesting that testicular androgen increases Aire expression in males. In addition, we compared Aire mRNA levels in thymic stroma of testicular feminized (AR^Tfm^/Y) mice versus WT male littermate controls. AR^Tfm^/Y mice have a spontaneously occurring, inactivating AR mutation, and therefore are insensitive to androgen. Aire mRNA levels were lower in thymic stroma of AR^Tfm^/Y mice compared with WT male littermate controls (Fig. 2g), suggesting that AR is important in increasing Aire expression in male mice. In addition, mRNA levels of two Aire-regulated TSAs, insulin and Spt-1 (ref. 6), were also lower in thymic stromal cells of AR^Tfm^/Y mice (Fig. 2h). AR effect was specific to Aire-regulated TSAs, since expression of Silver, an Aire-independent TSA^23^, was unchanged between the two strains. Together, these findings suggest that AR-dependent testicular androgen action is important in increasing Aire expression in male mice.

### DHT increases Aire expression in mouse and human thymus

**Figure 3:**
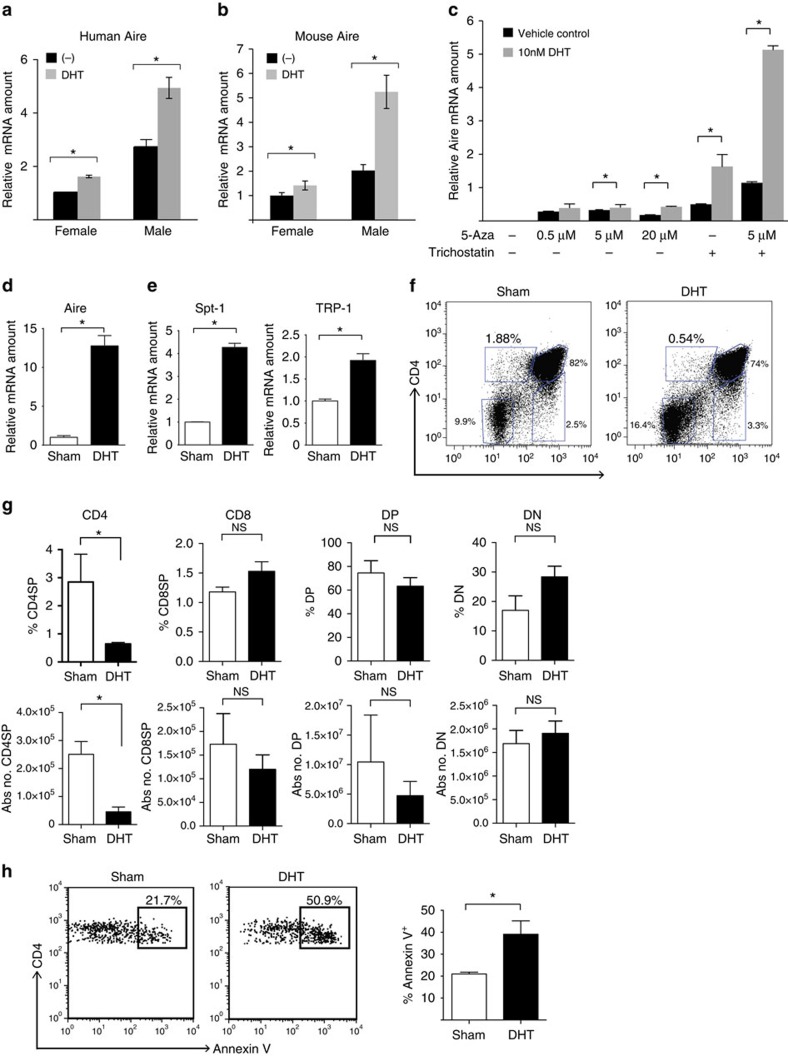
Androgen enhances Aire transcription, TSA expression and negative selection of self-reactive T cells. (**a**) Relative AIRE mRNA expression levels in male and female thymic stromal cells incubated with 10 nM DHT or vehicle control (−) for 6 h. Cells were from <1-week-old paired human subjects. Data shown are representative of at least three representative experiments. (**b**) Relative Aire mRNA expression levels in thymic stroma of 6-week-old male and female mice incubated with 10 nM DHT or vehicle control (−) for 6 h. Four mice were pooled per group, and data shown are representative of at least two independent experiments. (**c**) Relative Aire mRNA expression in LNCaP cells pretreated with indicated concentrations of 5-Aza and 100 ng ml^−1^ trichostatin in the presence of 10 nM DHT (gray bars) or vehicle control (black bars) for 8 h. Data shown are representative of two independent experiments. (**d**,**e**) Relative Aire (**d**) and TSA (**e**) (Spt-1 and TRP-1) mRNA expression in isolated thymic stroma from mice treated with DHT pellets or sham implant procedure. (**f**) Two-week-old TRP-1 TCR Tg RAG^−/−^ male mice were treated with 20 mg kg^−1^ acyline to minimize endogenous androgen production and implanted with DHT pellets or sham treated for 4 weeks total. Representative flow cytometry plots of thymocytes with frequencies of CD4 single-positive (CD4SP), double-positive (DP), CD8 single-positive (CD8SP) and double-negative (DN; clockwise, starting from left) populations shown in DHT-treated versus sham-operated male mice. (**g**) Average frequencies (top) and absolute numbers (bottom) of thymocyte subpopualtions as indicated for DHT-treated (*n*=4) and sham-operated (*n*=6) male mice. (**h**) Flow cytometric analysis of annexin V levels in CD4 single-positive thymocytes. Representative flow plot (left) and cumulative data (right) are shown. For all data sets, unpaired Student’s *t*-test was used for comparisons. Error bars represent s.e.m. *P<0.05. NS, not significant.

Intrigued by these findings, we tested whether androgen upregulates Aire expression *in vitro*. We compared Aire expression in primary thymic stromal cells cultured for 6 h with dihydrotestosterone (DHT), a potent testosterone metabolite that is not subject to conversion to oestradiol, or vehicle control. DHT induced a significant increase in Aire mRNA expression in human thymic stromal cells from both male and female subjects (Fig. 3a), with a larger effect in cells of males compared with females. Similar increases in Aire mRNA expression were seen with mouse thymic stromal cells, with a more pronounced effect with male cells (Fig. 3b). Finally, the addition of flutamide, an AR antagonist, blocked DHT induction of Aire in thymic stromal cells of male mice, which suggests that DHT upregulation of Aire expression was AR dependent (Supplementary Fig. 1).

In addition, we determined the effect of androgen on LNCaP cells, an epithelial cell line that expresses endogenous AR. LNCaP cells do not express Aire at baseline but can be induced to express Aire with 5-aza-2′-deoxycitidine (5-Aza), a demethylating agent, and trichostatin, a histone deacetylase inhibitor (Supplementary Fig. 2), as previously described^26^,^27^. Addition of 10 nM DHT further increased Aire mRNA expression (Fig. 3c), suggesting that androgen promotes Aire transcription in this cell line.

To determine the androgen effects on Aire expression *in vivo*, we implanted 2-week-old male C57BL/6 WT mice with DHT pellets for 2 weeks or subjected mice to a sham implantation. By quantitative RT–PCR analysis, DHT treatment increased Aire mRNA expression of thymic stromal cells compared with sham treatment (Fig. 3d). Furthermore, DHT treatment also increased mRNA expression of two Aire-regulated TSAs, Spt-1 and TRP-1 (refs 6, 23; Fig. 3e). Because Aire expression was compared using thymic stromal cells, DHT could either be increasing the proportion of mature mTECs that express Aire or directly increasing Aire expression within mature mTECs. To resolve whether DHT might be altering thymic epithelial cell (TEC) subset numbers, we subjected 3-week-old male mice to sham procedure or DHT pellet insertion and determined the frequency and absolute number of thymic epithelial subsets by flow cytometry 1–3 weeks later. No differences were seen in the frequency of mTECs (%CD45− MHCII+ Ly51^low^), cortical TECs (%CD45− MHCII+ Ly51^high^), mTEC^hi^ (%CD45− MHCII^high^ Ly51^low^) or mTEC^lo^ (%CD45− MHCII^low^ Ly51^low^; Supplementary Fig. 3a,b). Furthermore, the ratio of mTEC^hi^ to mTEC^lo^ was unchanged in sham- and DHT-treated mice (Supplementary Fig. 3c), suggesting that DHT is not increasing the proportion of mature MHCII^hi^ mTECs.

Of note, the absolute numbers of mTEC^hi^ and cortical TEC populations were significantly decreased with DHT treatment, and similar trends were seen with total mTEC and mTEC^lo^ populations, although these differences did not quite reach statistical significance (Supplementary Fig. 3d). Together, these findings suggest that DHT treatment has a global effect on stromal cell numbers, without disproportionately affecting a particular subpopulation. Thus, it is unlikely that the increased Aire expression seen in DHT-treated mice is due to an increased frequency of the differentiated mTEC subset but instead is due to increased Aire expression in mTECs. In line with this, DHT treatment increased Aire and Spt-1 mRNA expression in sorted mTEC^hi^ cells (Supplementary Fig. 4). Together, these findings provide evidence that DHT increases Aire and TSA expression in mTECs.

Given the increased TRP-1 expression in DHT-treated males, we asked whether DHT treatment would result in more efficient negative selection of TRP-1-reactive T cells. We utilized TRP-1 T-cell receptor (TCR) transgenic (Tg) mice to test this, since CD4 single-positive (SP) thymocytes in these mice normally undergo Aire-dependent negative selection^23^,^28^,^29^. Two-week-old TRP-1 TCR Tg male mice were treated with acyline, a gonadotropin-releasing hormone antagonist, to minimize endogenous androgen production^30^, then implanted with DHT pellets for 4 weeks or subjected to sham implantation. Representative flow plots with the frequencies of CD4SP, double-positive, CD8SP and double-negative cells among thymocytes are shown in Fig. 3f. With sham treatment, 1.88% of thymocytes were CD4SP cells in Aire-sufficient (WT) mice. This low frequency reflects Aire-dependent TRP-1 TCR Tg thymocyte negative selection without the addition of exogenous DHT. With DHT treatment, the CD4SP frequency further decreased to 0.54% suggesting that DHT enhances Aire-mediated negative selection. Quantification of frequency of CD4SP cells (*n*=4 for sham, *n*=6 for DHT treated) is shown in Fig. 3g. DHT treatment also decreased the absolute numbers of CD4SP cells. No significant differences were seen in double-negative, double-positive and CD8SP subpopulations (Fig. 3f,g). Consistent with increased clonal deletion, increased frequency of apoptotic, annexin V-positive CD4SP thymocytes was seen in DHT-treated mice (Fig. 3h). An alternative explanation to consider is that DHT is directly toxic to thymocytes. This possibility was tested by incubating thymocytes for 6 h in either vehicle control or 10 nM DHT. The frequency of annexin V+ cells was not different in the two groups, which suggests that DHT is not acting directly on thymocytes (Supplementary Fig. 5). A caveat to this experiment, however, is that apoptosis was compared in a short-term *in vitro* culture system, which may not reflect the thymocyte environment *in vivo*. Additional evidence that DHT is not directly acting on thymocytes is provided by a previous report utilizing haematopoietic chimeras in which AR is expressed only on haematopoetic cells and not in the non-haematopoietic cells, such as TECs^31^. DHT treatment of these chimeras did not alter the frequencies of thymocytes subsets, suggesting that AR expression on thymocytes does not affect thymocyte development. Together, these data suggest that androgen enhances Aire expression in mTECs, with subsequent increase in TSA expression and T-cell negative selection.

### Androgen activation targets AR to the Aire promoter

**Figure 4:**
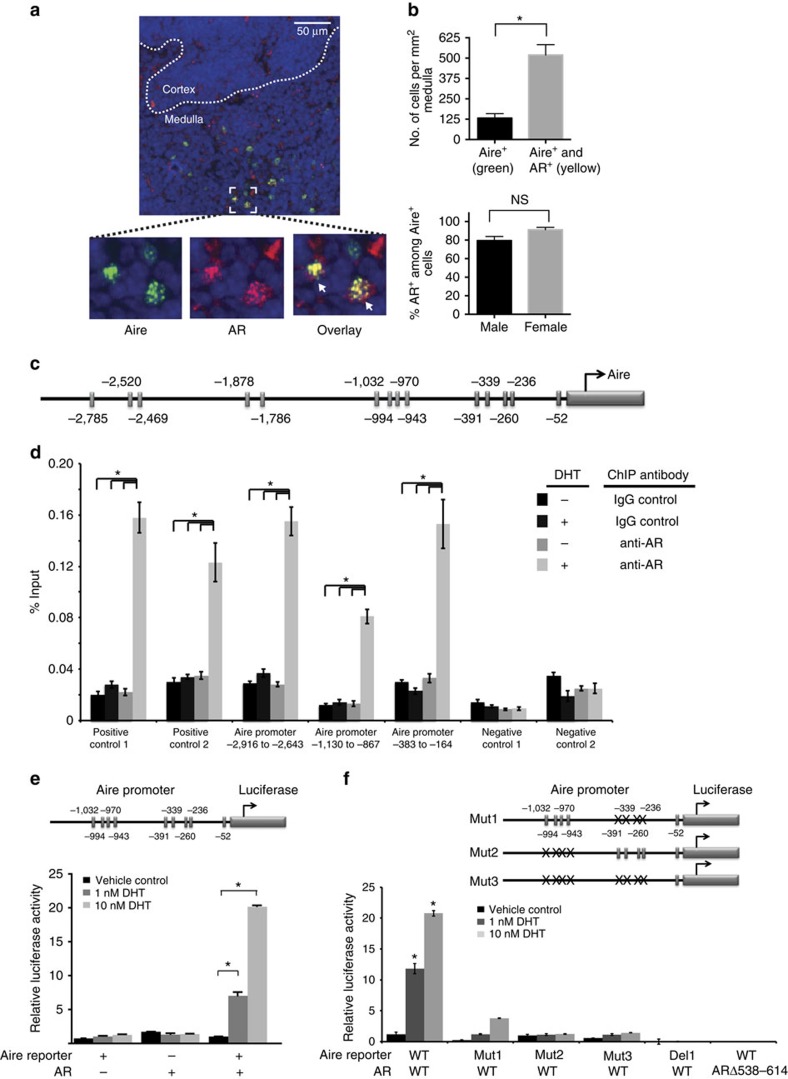
Androgen activates AR to target to the Aire promoter. (**a**) Top: representative immunofluorescence staining of frozen thymic section from an 8-week-old male mouse. Slides were scanned at × 20 magnification, with cortex and medulla delineated by a white dotted line. Bottom: expansion of bracketed area to focus on Aire (green), AR (red) and 4,6-diamidino-2-phenylindole nuclear (blue) staining. Arrows indicate cells co-expressing Aire and AR. (**b**) Top: quantification of cells per thymic medulla area that are Aire^+^ (green) and Aire^+^+AR^+^ positive (yellow). DAPI delineates cell nuclei. Bottom: quantification of AR co-expression within Aire^+^ cells in males and females. Data shown are averages+s.d. of four 0.04-mm^2^ areas. (**c**) Schematic diagram of 14 predicted AR-binding sites (rectangles; numbers indicate position relative to Aire translational start site) within a 2.8-kb human AIRE gene 5′ flanking region. (**d**) AR chromatin immunoprecipitation (ChIP) in LNCaP cells pretreated with 5 μM 5-Aza and 100 ng ml^−1^ trichostatin. Cells were treated with 10 nM DHT (+) or vehicle control (−). Quantitative PCR for AR or IgG control ChIP was performed over two positive control regions (prostate-specific antigen promoter and enhancer)and two negative control regions (−1,575 to −1,275 region upstream of Aire translational start site (1) and Aire exon (2)). Three Aire 5′ flanking regions containing predicted AR-binding sites were tested for AR accumulation. Numbers indicate base pairs upstream of Aire translational start site. Data shown are representative of three independent experiments performed. (**e**) AIRE promoter activity measured by luciferase. HEK293T cells were transiently transfected with pAP1235 human AIRE promoter–luciferase reporter plasmid and/or pCMV-AR in the presence of the indicated DHT concentrations for 24 h. Data shown are representative of two independent experiments. (**f**) AIRE promoter activity, as in **e**, but with four mutant AIRE promoter–luciferase constructs (Mut1, Mut2, Mut3 and Del) that alter predicted AR-binding sites in the 5′ AIRE flanking region (Supplementary Table 1). AR expression vector pCMV-ARΔ538–614 with a deletion of the AR DNA-binding domain (binding Mut) was also tested. For all data sets, unpaired Student’s *t*-test was used for comparisons. Error bars represent s.e.m. *P<0.05. NS, not significant.

We next sought to determine the molecular mechanism by which androgen increases Aire expression in the thymus. AR is expressed in the thymus, with highest levels in TECs^31^,^32^. More specifically, AR is expressed in multiple TEC subsets, including the MHCII^high^ mTEC (mTEC^hi^) population^33^. As noted above, mTEC^hi^ cells are of special interest because Aire-expressing (Aire^+^) mTECs are highly enriched in this subset^20^. To verify that AR protein is expressed by Aire^+^ cells, we performed immunofluorescent staining of mouse thymic tissue for AR and Aire. As expected, Aire was restricted to the nucleus of thymic medullary cells (Fig. 4a). AR was present in the nucleus as well as the cytoplasm of thymic medullary cells (Fig. 4a), and most Aire^+^ cells co-expressed AR (Fig. 4a,b). A high degree of AR and Aire co-expression was noted in thymic medulla of male and female mice (Fig. 4b). Given these data (Fig. 4a; ref. 33), we hypothesized that androgen/AR complexes may directly regulate Aire transcription through their interaction with the Aire promoter.

Analysis of the human AIRE gene 5′ flanking region using PROMO software revealed 14 potential AR-binding elements in a 2.8-kb region (Fig. 4c). To determine whether androgen/AR complex accumulates at Aire promoter regions, we performed AR chromatin immunoprecipitation in LNCaP cells. As described previously (Fig. 3c), LNCaP cells endogenously express AR and can be induced to express Aire with 5-Aza and trichostatin^26^,^27^. In the presence of DHT, AR was selectively enriched at three Aire promoter regions that were predicted to contain AR-binding sites (position −2,916 to −2,643, −1,130 to −867 and −383 to −164; Fig. 4d). These findings suggest that androgen activation results in AR accumulation at the Aire promoter.

We next utilized a reporter system in human embryonic kidney 293T (HEK293T) epithelial cells to delineate further how androgen interacts with the Aire promoter. HEK293T cells were transfected with pAP1235, an Aire reporter construct comprised of a human AIRE gene 5′ flanking region (position −1,235 to +1 relative to translational start) in front of luciferase^26^. This 5′ flanking region is sufficient to drive Aire transcriptional activity^28^ and contains nine predicted AR-binding elements (Fig. 4e). An AR expression construct was also transfected into HEK293T cells because HEK293T cells do not express endogenous AR^34^. In co-transfected HEK293T cells, DHT strongly induced AIRE promoter activity in a dose-dependent manner (Fig. 4e). Importantly, mutagenesis of predicted AR-binding sites in the Aire 5′ flanking promoter region abolished AIRE promoter activity (Fig. 4f; Supplementary Table 1). These findings suggest that androgen/AR-mediated Aire upregulation requires the presence of AR-binding sites. Moreover, transfection of a mutant AR construct (ARΔ538–614)^35^ in which AR’s DNA-binding region is deleted also abolished AIRE promoter activity. Thus, AR’s DNA-binding region is required to promote Aire expression. Together, these findings suggest that androgen/AR complexes may directly interact with the Aire promoter to upregulate Aire transcription.

### Androgen decreases EAE severity in an Aire-dependent manner

Since DHT upregulates Aire expression, we tested whether androgen upregulation of Aire may protect against autoimmune disease. We utilized the experimental autoimmune encephalitis (EAE) mouse model of multiple sclerosis that can be induced by active immunization with Myelin oligodendrocyte glycoprotein (MOG)_35–55_ peptide. MOG EAE was attractive as an experimental system because androgen administration has been shown to ameliorate the severity of MOG EAE^4^. Whether the protective effect of androgen is dependent on Aire, however, is not known.

**Figure 5:**
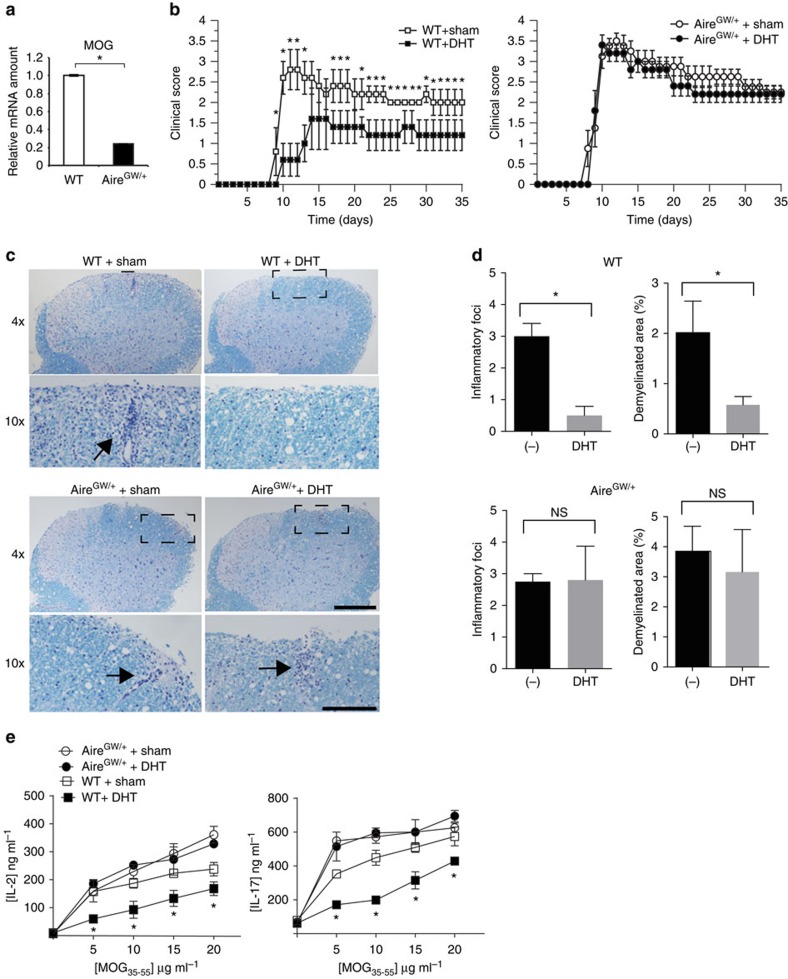
Androgen protects against EAE in an Aire-dependent manner. (**a**) Relative MOG mRNA expression in sorted WT and Aire-deficient (Aire^GW/+^) mTECs was determined by quantitative RT–PCR. Four mice were pooled per group, and data shown are representative of at least two independent experiments. (**b**) Average clinical EAE scores of WT (left) or Aire^GW/+^ (right) male mice implanted with DHT pellets or sham operated following immunization with MOG_35−55_ peptide. *n*=5 in each group. Shown is representative of three independent experiments. (**c**) Representative Luxol Fast Blue-PAS-stained lumbar spinal cord sections from WT (top panels) or Aire-deficient Aire^GW/+^ (bottom panels) mice treated with placebo (sham) or DHT pellet collected 35 days after MOG immunizations (*n*≥5 for each group). Box in × 4 image indicates area shown in × 10 image. Arrows indicate inflammatory foci with demyelination. Scale bar, 200 μm (× 4); 100 μm (× 10). (**d**) Mean numbers of inflammatory foci and demyelinated area in WT and Aire^GW/+^ mice treated with placebo (−) or DHT pellet. (**e**) Concentrations of IL-2 and IL-17 cytokine in supernatants of splenocytes cultured in the presence of increasing amounts of MOG_35−55_ peptide. Splenocytes were isolated from WT or Aire^GW/+^ mice treated with DHT or sham at day 35 after EAE induction. Unpaired Student’s *t*-test was used in all data sets, except for survival curves in which Mann–Whitney *U*-test was utilized. Error bars represent s.e.m. *P<0.05. NS, not significant.

We hypothesized that androgen protects from MOG EAE by upregulating Aire expression, which subsequently increases thymic MOG expression to enforce deletion of MOG-reactive T cells. To test this hypothesis, we first verified that Aire regulates MOG antigen expression in the thymus by comparing MOG expression in sorted mTECs from Aire-deficient versus WT mice. Aire-deficient mice, harbouring a dominant Aire G228W mutation (Aire^GW/+^ mice)^12^, expressed less MOG in mTECs than WT controls (Fig. 5a). This finding confirmed a previous report that Aire controls MOG expression in the thymus^36^. Furthermore, incubation of thymic stroma from WT male mice in 10 nM DHT induced MOG mRNA expression (Supplementary Fig. 6).

To test whether androgen protection is Aire dependent, we treated male WT and Aire^GW/+^ mice with DHT or vehicle control for 4 weeks before immunization with MOG_35−55_ peptide. As expected^4^, DHT-treated WT mice had milder clinical disease than sham-treated controls (Fig. 5b, left; Supplementary Table 2). In contrast, no differences in clinical EAE severity were seen in DHT-treated or sham-treated Aire^GW/+^ mice (Fig. 5b, right; Supplementary Table 2), suggesting that the protective effect of androgen is Aire dependent.

In WT mice, DHT administration was associated with decreased inflammatory foci and demyelination in the lumbar spine (Fig. 5c,d, top panels); in Aire^GW/+^ mice, on the other hand, DHT treatment did not have an effect (Fig. 5c,d, bottom panels). In addition, DHT administration decreased splenocyte interleukin-2 (IL-2) and IL-17 inflammatory cytokine secretion in WT mice (Fig. 5e). In contrast, DHT treatment did not have an effect in Aire^GW/+^ mice (Fig. 5e). Together, these findings suggest that the protective effect of DHT on MOG-induced EAE requires Aire. Of note, our data does not rule out that androgen decreases EAE severity through an indirect, Aire-mediated mechanism. For instance, since Aire affects thymic regulatory T-cell (Treg) development^8^, androgen may promote Treg suppressive function in WT (Aire sufficient) mice without affecting thymic Tregs in Aire-deficient mice.

### Male gender protects against EAE in an Aire-dependent manner

**Figure 6:**
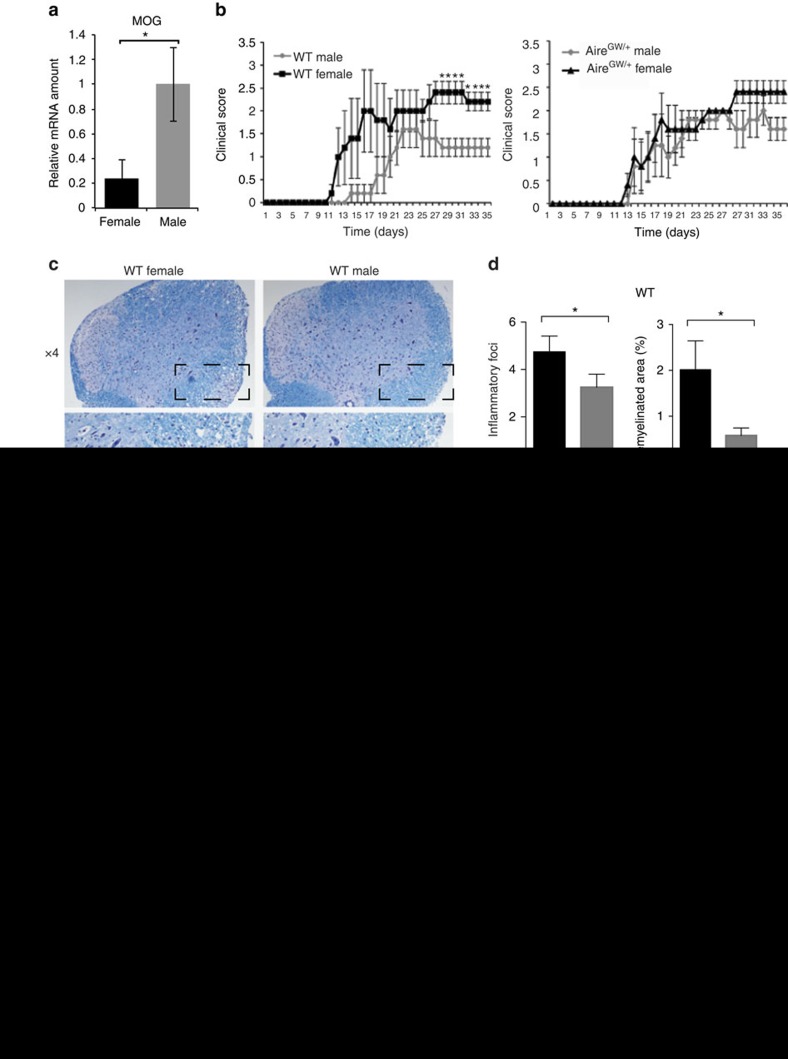
Male gender protects against EAE in an Aire-dependent manner. (**a**) Relative MOG mRNA expression amounts in sorted mTECs from female and male wild-type (WT) mice by quantitative RT–PCR. Four mice were pooled per group, and data shown are representative of at least two independent experiments. (**b**) Mean clinical EAE scores of male versus female WT or Aire-deficient (Aire^GW/+^) mice following immunization with MOG_35−55_ peptide. (**c**) Representative Luxol Fast Blue-PAS-stained lumbar spinal cord sections from male versus female WT (top panels) or Aire^GW/+^ (bottom panels) mice collected 35 days after MOG immunizations (*n*≥5 for each group). Box in × 4 image indicates area shown in × 10 image. Arrows indicate inflammatory foci with demyelination. Scale bar, 200 μm (× 4) and 100 μm (× 10). (**d**) Mean numbers of inflammatory foci and demyelinated area in male versus female WT and Aire^GW/+^ mice. (**e**) Concentrations of IL-2 and IL-17 cytokine in the supernatants of splenocytes cultured in the presence of increasing amounts of MOG_35−55_ peptide. Splenocytes were isolated from male and female WT or Aire^GW/+^ mice at day 35 after EAE induction. Unpaired Student’s *t*-test was used in all data sets, except for survival curves in which Mann–Whitney *U*-test was utilized. Error bars represent s.e.m. *P<0.05. NS, not significant.

Our data suggest that male gender is associated with increased Aire and TSA expression in the mouse thymus (Figs 1c and 2a–c). In line with this, sorted mTECs from male C57BL/6 WT mice expressed increased MOG mRNA compared with female mice (Fig. 6a). Given this increased MOG expression in male versus female thymus, we next asked whether male gender also protects against EAE in an Aire-dependent manner. In WT mice, clinical disease was more severe in females compared with males (Fig. 6b, left; Supplementary Table 3). In Aire-deficient (Aire^GW/+^) mice, on the other hand, clinical disease was not different between the two groups (Fig. 6b, right; Supplementary Table 3), suggesting that amelioration of autoimmunity in males required Aire.

In WT mice, male gender was associated with decreased the numbers of inflammatory foci and the areas of demyelination in the lumbar spine (Fig. 6c,d, top). In Aire^GW/+^ mice, in contrast, these differences were not seen. Finally, male gender was associated with decreased splenocyte inflammatory cytokine production in WT mice (Fig. 6e). These differences were abolished in Aire-deficient Aire^GW/+^ mice (Fig. 6e). Together, these results suggest that male gender is protective against MOG-induced EAE and that this protection is dependent on Aire.

## Discussion

Gender bias in multiple sclerosis and other autoimmune diseases is well-documented (reviewed in ref. 5). What underlies these gender differences, however, is not clear. Gender differences in autoimmunity have been attributed to multiple factors, including differences in sex steroid hormone levels. In particular, increased androgen levels are associated with protection from autoimmunity, although the mechanism(s) that confer this protection are not clear. In this study, we provide evidence that increased androgen levels in males may protect from autoimmunity by maintaining higher levels of thymic Aire expression (Supplementary Fig. 7). By ‘dialing up’ the level of Aire, androgen also upregulates Aire-mediated TSA expression and negative selection of self-reactive T cells. Androgen therefore reinforces a central tolerance barrier, which limits the release of autoimmune T cells into the periphery.

The mechanisms by which androgen protects against autoimmunity have been the subject of intense interest. Androgen has been proposed to avert autoimmunity by upregulating TGF-beta production in thymic stromal cells^37^. Recently, two studies have reported that androgen regulation of gut microbiota contributes to gender differences in autoimmunity predisposition in mice^2^,^3^. Androgen regulation of bone marrow stromal cells and subsequent transforming growth factor-beta (TGF-beta) production has also been proposed as a mechanism by which androgen protects against autoimmunity^31^. In line with this, androgen may also exert anti-inflammatory effects by decreasing inflammatory cytokine and growth factor production by prostate stromal cells^38^, and by repressing transcription factors important in peripheral immune cell activation^39^. Our finding that androgen regulates Aire expression in mTECs represents a distinct pathway for androgen-mediated protection against autoimmunity, which is not mutually exclusive of the previously reported mechanisms. It has been reported that Aire is required early, but is dispensable later in life^40^. Early in life, infants also undergo ‘mini-puberty’, a period in which male infants produce pubertal levels of androgen. This temporal overlap suggests the possibility that the neonatal androgen surge may promote Aire expression at a time when Aire function is critical, thus ensuring protection from autoimmune disease development.

Androgen is known to have multiple effects on the thymus. Castration of male mice is associated with thymic enlargement, whereas androgen replacement reduces thymic size^41^. Interestingly, hypogonadal men have increased thymic output of T cells, which is reversed with androgen administration^42^. Furthermore, androgen administration has been associated with increased thymocyte apoptosis^43^,^44^. These findings are consistent with our findings that androgens enhance thymocyte negative selection by upregulating Aire expression and peripheral self-antigen expression. Whether increased negative selection accounts for all or part of the increased thymocyte apoptosis seen in androgen-treated mice remains to be determined. In the setting of thymic injury, such as bone marrow transplantation (BMT), androgen depletion has been reported to protect TECs and promote subsequent T-cell immune reconstitution. For instance, androgen blockade and keratinocyte growth factor in combination protects TEC subsets from depletion during BMT, suggesting that androgen blockade may have cytoprotective effects in the setting of thymic injury^45^. Androgen blockade also enhanced the efficacy of BMT as a tolerizing strategy for EAE. This enhancement appeared to be mediated by androgen’s promotion of thymic regeneration and subsequent immune reconstitution^46^. Thus, in the setting of BMT, androgen appears to play an important role in cytoprotection and regeneration of TECs.

We propose a model in which androgen quantitatively upregulates Aire expression, and that androgen levels in males may increase Aire expression to a degree that protects against autoimmunity. Such a quantitative model of androgen effects leaves open the possibility that other factors may contribute to protection against autoimmunity in males. Furthermore, it should be noted that Aire-deficient female mice, compared with male mice, continue to develop certain autoimmune diseases at an increased incidence. Thus, other sex-associated factors are likely to influence autoimmune disease incidence or severity in an Aire-independent manner. In addition to androgen, other hormones also vary between males and females, and may contribute to differences in autoimmune susceptibility. Higher oestrogen levels, for example, may increase autoimmune susceptibility in females. Oestrogen has been reported to have multiple immune effects, including altering CD4 to CD8 T-cell ratios, increasing B-cell survival and increasing B-cell antibody production, depending on the concentration and other factors^47^. Similarly, differences in progesterone levels may also alter immune function in females versus males^48^. Interestingly, a recent study reported that the mTEC transcriptome is not different between male and female mice^49^, a finding which seems to conflict with our results. Furthermore, in this study, mTECs from castrated males expressed increasesd Aire-dependent TSAs, with similar levels of Aire, compared with intact mice. A potential explanation for this discrepancy may be the age of the mice; the latter study utilized 24-week-old mice, whereas we studied 6–8-week-old mice. Thus, the contributions of sex hormones to autoimmune susceptibility are likely multiple and complex. Whether additional hormones besides androgens may impinge on Aire expression to alter immune function, and the effect of age on these alterations, remains to be determined.

Aire prevents autoimmunity by promoting the transcription of peripheral TSAs within the thymus and negative selection of self-reactive T cells^6^. In the general population, Aire mRNA levels vary greatly between individuals and are strongly correlated with TSA expression in the thymus^13^. What determines Aire transcription levels, however, is not completely understood. To date, putative binding sites for Sp1, NF-Y and AP-1 transcriptional complexes within the Aire promoter have been identified^26^. In addition, the Ets family of transcription factors has been implicated in positive regulation of Aire transcription^50^. Our studies provide evidence that androgen/AR complexes also associate with the Aire promoter to upregulate Aire promoter activity. Interestingly, members of the Ets family of transcription factors have been reported to interact with AR in regulating transcriptional activity^51^,^52^. Thus, it is plausible that androgen/AR complexes interact with Ets family members or other transcription factors in mTECs to control Aire transcription. An important caveat to keep in mind is that a subset of our experiments was performed in non-mTEC (LNCaP and HEK293) cell lines. This concern is tempered by the use of transfected HEK293 cells to understand mechanisms of Aire function^53^,^54^.

In addition to promoting thymic negative selection of autoreactive thymocytes through TSA upregulation, Aire also prevents autoimmunity through additional mechanisms. For instance, several studies have suggested that Aire may promote thymic negative selection through TSA-independent mechanisms^9^,^55^. In particular, Aire may regulate processing or presentation of TSAs by thymic antigen-presenting cells or expression of chemokines important in thymic dendritic cell recruitment. In addition, Aire plays a crucial role in thymic development of regulatory T cells^8^, a T-cell subset with potent immunoregulatory function. Finally, Aire is also expressed in peripheral lymphoid organs and may upregulate a complementary set of TSAs to enforce peripheral tolerance^56^. It is currently not clear whether androgen modulation of Aire expression also affects these additional tolerance mechanisms, and this question will be the subject of future investigations.

At this time, compounds that promote Aire expression as potential therapies for autoimmunity do not exist. Our study suggests that androgen therapy may directly enhance Aire expression to protect against autoimmunity. Indeed, preliminary studies have demonstrated androgen efficacy in multiple sclerosis^57^,^58^, an autoimmune disease in which Aire regulates thymic expression of the antigenic target MOG. Our study also suggests that developing strategies to target androgen to the thymus may provide benefit for autoimmunity while minimizing virilizing side effects. Thus, findings from this study may help direct the use of androgen in the treatment of autoimmunity.

## Methods

### Mice

AR^Tfm^/Y mice on C57BL/6 background (JAX 001809), aged 4–8 weeks, were purchased from the Jackson Laboratory, Bar Harbor, Maine. Male and female WT mice and Aire-deficient (Aire^GW/+^) littermates^12^ on C57BL/6 background, aged 6–12 weeks, were bred in a specific pathogen-free mouse colony. TRP-1 TCR Tg RAG^−/−^ mice harbour a transgene encoding a TCR specific for TRP-1 in the context of MHCII on a recombinase-activating gene-deficient background^28^,^59^. Male Tyrp1^B-w^ TRP-1 TCR Tg RAG^−/−^ mice on C57BL/6 background (JAX 008684), aged 6–12 weeks, were purchased from the Jackson Laboratory, Bar Harbor, Maine, and bred to female RAG^−/−^ mice on C57BL/6 background, aged 6–12 weeks, in a specific pathogen-free mouse colony to generate TRP-1 TCR Tg RAG^−/−^ male mice with WT Tyrp1 gene, so that endogenous TRP-1 protein is expressed. C57BL/6J (Stock 000664 JAX) male and female mice were purchased at 6–8 weeks of age. Mice were used in accordance with guidelines from the University of North Carolina at Chapel Hill Animal Care and Use Committee. Experiments were conducted with the approval of the Animal Use and Care Committees of the University of North Carolina at Chapel Hill.

### Androgen treatment *in vivo*

Androgen treatment was performed using DHT pellets or subcutaneous DHT injections. DHT pellets provide 0–10 mg per 60 day release (Innovative Research of America) and were implanted subcutaneously into 2-week-old TRP-1 TCR Tg RAG^−/−^ on C57BL/6 background male mice as per manufacturer’s instruction. Circulating DHT levels after implantation are shown in Supplementary Fig. 8. Alternatively, DHT (Sigma) was dissolved in vegetable oil (40 mg per 0.1 ml per mouse) and injected subcutaneously. For sham-operated controls, mice were subjected to the same procedure except those that were not administered a DHT pellet. For placebo pellet controls, mice were subjected to the same procedure and administered a placebo pellet.

### Primary human and mouse thymus cell culture

Human neonatal thymus tissue was obtained as surgical discards from cardiac surgery under protocols approved by the Institutional Review Board at University of North Carolina at Chapel Hill. Thymus was subjected to mechanical disruption, 0.125% collagenase/dispase and 0.1% DNase I digestion, and agitated with a Pasteur pipette continuously at 37 °C for 30 min to break the clumps^60^. The fragments were then run on a Percoll density gradient to separate the thymocytes and the TEC stromal fraction. The stromal fraction was incubated in DMEM (Gibco) with 10% charcoal-stripped serum for 6 h in the presence of 10 nM DHT or vehicle control. For inhibitor studies, flutamide (5 μM) or vehicle control was added to cultures for 2 h before 6-h incubation with 10 nM DHT or vehicle control.

### Quantitative RT–PCR

RNA was prepared from stromal fractions or flow-sorted cell fractions using the RNeasy Mini Kit (Qiagen) and reverse transcribed to complementary DNA. For a portion of human thymus specimens, fragments of thymus tissue were flash-frozen before RNA preparation. Quantitative RT–PCR was performed using previously reported primer/probe sets for Aire and insulin^60^. Primer/probe sets for MOG (Mm00442688_m1), Trp-1 (Mm00453202_m1), tyrosinase (Mm00495817_m1), Silver (Mm00498996_m1), Aire (Hs00230829_m1), AR (Mm00442688_m1; Hs00171172_m1), KRT5 (Hs00361185_m1), Krt10 (Mm03009921_m1), Csn2 (Mm04207885_m1), Gad1 (Mm04207432_g1), Resp18 (Mm00485697_m1), Fabp9 (Mm01964336_s1), Obp1a (Mm00500903_m1), Reg3b (Mm00440616_g1) and 18s (Hs99999901_s1) were purchased from Applied Biosystems. Quantitative RT–PCR was performed using a 7900HT Fast Real-Time PCR System (Applied Biosystems). The housekeeping genes peptidylprolyl isomerase A (PPIA, cyclophilin A) or KRT5 were used as an internal control, since mRNA levels of PPIA and KRT5 are androgen independent^11^,^61^. The standard curve method was used to analyse target gene expression.

### RNA sequencing of mTECs

Libraries (3.5 × 10^6^ to 13.5 × 10^6^ mapped reads per sample) were generated from sorted mTECs (PI^−^ CD45^−^ MHCII^+^ Ly51^low^) isolated from the TEC fraction, as described above with some modifications. Ten mice were pooled per biologic replicate, with two biologic replicates per group (male versus female). mTECs were pelleted, lysed in RLT lysis buffer (Qiagen) and total RNA was recovered using the RNeasy Micro Kit (Qiagen). RNA quality and concentration were assessed using the Experion high-sensitivity RNA analysis kit (Bio-Rad). Complementary DNA libraries were prepared using the SMARTer Ultra Low kit v3 (Clontech). Fifty-base pair reads were sequenced on an Illumina HiSeq 2000 through the UNC High Throughput Sequencing Core. Low-quality reads were removed using Trimmomatic^62^ and the remaining reads were aligned to the mm9 assembly of the mouse genome with the TopHat2 splice-junction mapper^63^. Reads were assigned to genes using HTseq-count^64^. The normalized fragment counts per transcript, RPKM (reads per kilobase of transcript per million mapped reads), and the log fold changes were calculated by edgeR^65^. The heatmap was generated by combining our RPKM values with those from the mature Aire-positive and mature Aire knockout mTEC replicates from ref. 21 (GEO accession number GSE53111). The list of 3,980 Aire-regulated genes was downloaded from the Supplementary Data that accompanied ref. 21. Of these, 2,760 (70%) were detected in our RNA sequencing experiments and used in the heatmap (GEO accession number GSE79014).

### Antibodies and flow cytometry

Antibodies used in this study were purchased from eBioscience (CD4 (RM4–5)—catalogue no. 12-0042 used at 0.125 μg per test; CD3 (145-2c11)—catalogue no. 250031 used at 1 μg per test; and Annexin V catalogue no. 17–8007 used at 5 μl per test). CD8 (5H10)—catalogue no. MCD0830 used at 0.1 μg per test was purchased from ThermoFisher Scientific. Vβ14 (14-2)—catalogue no. 553258 used at 1 μg per test was purchased from BD Biosciences. Cells were analysed on a Dako CyAn flow cytometer (Beckman-Coulter) using FlowJo Software (TreeStar).

### Immunofluorescence staining

Frozen thymic sections (8 μm) were acetone fixed and immunostained as described^12^. Aire antibody (5H12; eBioscience used at 2.1 μl per 100 μl dilution solution), K5 antibody (Ab 53121; Abcam used at 0.001 μl per 100 μl dilution solution) and AR antibody (Ab 74272; Abcam used at 0.35 μl per 100 μl dilution solution) staining was detected with biotinylated goat anti-rat light chain (Millipore), phycoerythrin (PE)-conjugated donkey anti-rabbit IgG (eBioscience) secondary antibodies and tertiary streptavidin-conjugated fluorescein isothiocyanate (eBioscience). Slides were mounted in Dapi Fluoromount-G (Southern Biotech).

The Aperio FL digital pathology scanning system was used to scan each whole-mouse thymus section at a magnification of × 20. This system allows fluorescent whole-slide imaging of tissue sections at high resolution. Using ImageScope, the ratio of Aire+ cells to K5+ medulla area was calculated as follows: four 600 × 600-μm sections of K5+ areas were randomly selected with the scorer blinded to the presence Aire-positive cells in these areas. For each 0.36-mm^2^ region, the area of K5 was determined by tracing the outline of the medulla region, excluding areas of black empty space. The number of Aire-positive green cells was counted manually using the ImageScope Cell Counter tool. The average ratio of Aire+ cells to K5+ area within the four sections were calculated per mouse. *n*=4 mice per group.

Total mTEC cell numbers were determined by total counts obtained during flow cytometric sorting for mTECs. Gating for mTECs was performed per^20^. *n*=4 pooled mice in each group per replicate, with three replicates.

### Transient transfections and luciferase reporter assays

HEK293T cells (10^5^ cells per well in 24-well plates) were transiently transfected with 0.3 μg each of pAP1235 Aire promoter–luciferase reporter plasmid (a gift from Dr Part Peterson, University of Tampere, Estonia)^26^, Renilla control vector and/or human AR expression vector pCMV-AR^66^ using Lipofectamine 2000 (Invitrogen) according to the manufacturer’s instructions. After 48 h, luciferase activity measured using the Dual-Luciferase Assay System (Promega) as per the manufacturer’s instruction. Luciferase activity was normalized for transfection efficiency according to the Renilla luciferase activity. Transfections were performed in triplicate. Mutant Aire promoter DNA was synthesized by Genscript. Sequences of mutant AIRE promoter–luciferase constructs (Mut1, Mut2, Mut3 and Del) that alter predicted AR-binding sites in the 5′ AIRE flanking promoter region are shown in Supplementary Table 1. Mutant promoters were cloned into pGL4.70 vector (Promega) containing hRluc reporter gene and constructs verified by sequencing.

### Transcriptional activation assays

The metastatic human prostate cancer cell line LNCaP (ATCC) was maintained in DMEM with 10% fetal bovine serum. The DNA methyltransferase inhibitor 5-Aza was added to cells at final concentrations from 0.5 to 20 μM for 72 h. The deacetylase inhibitor tricostatin (0.1 μg ml^−1^) was added for 24 h alone or after 5-Aza treatment as indicated. Transcriptional expression of Aire was determined by quantitative RT–PCR.

### *In vivo* negative selection assay

Two-week-old TRP-1 TCR Tg RAG^−/−^ mice were injected with 20 mg kg^−1^ acyline (provided by NIH/NICHD) and implanted with DHT pellets for 4 weeks. Apoptosis of CD4SP thymocytes was determined by flow cytometry following annexin V staining.

### Experimental autoimmune encephalitis

DHT pellets were implanted in 6-week-old Aire-deficient Aire^GW/+^ mice and WT control male mice, 4 weeks before EAE induction. Mice were randomly assigned to the DHT or sham control groups. EAE was induced in 10-week-old Aire^GW/+^ mice and age-matched WT mice via subcutaneous immunization with 0.1 mg MOG p35–55 peptide in an emulsion with complete Freund’s adjuvant. Mice were injected intravenously with 50 ng Bordetella pertussis toxin at the time of and 2 days after immunization. Mice were examined daily for clinical signs of EAE and scored on a 5 point scale: 0, no clinical disease; 1, limp tail; 2, hindlimb weakness; 3, complete hindlimb paralysis; 4, hindlimb paralysis and some forelimb paralysis; and 5, moribund or dead. No blinding was done in this experiment.

### EAE histopathology

Mice were perfused with PBS and spinal cord was removed as described^67^. Lumbar spinal cords were formalin-fixed, embedded in paraffin, sectioned (5 μm) and stained with Luxol Fast Blue-PAS as described^68^. Quantification of inflammatory foci and areas of demyelination were performed as described^69^.

### Chromatin immunoprecipitation

Chromatin immunoprecipitation was performed as described^70^. LNCaP cells were grown in DMEM supplemented with 10% charcoal–dextran-stripped fetal bovine serum. After 2 days of culture, cells were treated with 5 μM 5-Aza and 100 ng ml^−1^ trichostatin for 24 h followed by 10 nM DHT treatment or vehicle control for 2 h. DNA and protein were crosslinked with 1% formaldehyde. Cells were lysed with 1% SDS, 10 mM EDTA, 50 mM Tris-HCl, pH 8.1, and protease inhibitor cocktail (Roche Molecular Biochemicals) and sonicated 12 times at 15 s each. Fragments of ∼200 bp were visualized. Supernatants were collected and diluted in 1% Triton X-100, 2 mM EDTA, 150 mM NaCl and 20 mM Tris-HCl, pH 8.1, followed by immunoclearing with 2 μg sheared salmon sperm DNA, 20 μl preimmune serum and protein A-Sepharose (45 μl of 50% slurry in 10 mM Tris-HCl, pH 8.1, and 1 mM EDTA) for 2 h at 4 °C. Immunoprecipitation was performed for 6 h or overnight at 4 °C with 5 μg of AR antibody (C-19, Santa Cruz) or control rabbit IgG (Santa Cruz). After immunoprecipitation, 45 μl protein A-Sepharose and 2 μg of salmon sperm DNA were added, and the incubation was continued for another hour. Sepharose beads were washed sequentially for 10 min each in TSE I (0.1% SDS, 1% Triton X-100, 2 mM EDTA, 20 mM Tris-HCl, pH 8.1, and 150 mM NaCl), TSE II (0.1% SDS, 1% Triton X-100, 2 mM EDTA, 20 mM Tris-HCl, pH 8.1, and 500 mM NaCl) and buffer III (0.25 M LiCl, 1% NP-40, 1% deoxycholate, 1 mM EDTA, and 10 mM Tris-HCl, pH 8.1). Beads were washed three times with TE buffer and extracted three times with 1% SDS, 0.1 M NaHCO_3_. Eluates were pooled and heated at 65 °C for 6 h or overnight to reverse the formaldehyde crosslinking. DNA fragments were purified with a QIAquick Spin Kit (Qiagen) and analysed for the presence of Aire promoter DNA by quantitative PCR. The primers are listed in Supplementary Table 4. PCR conditions were 94 °C for 3 min, (94 °C 30 s, 60 °C 30 s and 72 °C 30 s) for 36 cycles, and 72 °C for 10 min.

### Statistics

PRISM 5.0 and Microsoft Office Excel software were used to analyse the data (GraphPad Software, Inc.). Unpaired two-tailed Student’s *t*-tests were used to compare differences between the two groups. Sample sizes were determined by power analysis based on estimated sample variances. Survival curves were compared by Mann–Whitney *U*-test. *P* values of ≤0.05 were considered significant. All data are expressed as the means±s.e.m., except where indicated.

## Additional information

**How to cite this article:** Zhu, M.-L. *et al*. Sex bias in CNS autoimmune disease mediated by androgen control of autoimmune regulator. *Nat. Commun.* 7:11350 doi: 10.1038/ncomms11350 (2016).

## Supplementary Material

Supplementary Information Supplementary Figures 1-8 and Supplementary Tables 1-4
