# Supplementary material for: Sex bias in CNS autoimmune disease mediated by androgen control of autoimmune regulator
Source: Nat Commun. 2016 Apr 13;7:11350. doi: 10.1038/ncomms11350 (PMC5512610; doi:10.1038/ncomms11350)
Supplement: Supplementary Information — Supplementary Figures 1-8 and Supplementary Tables 1-4 [file ncomms11350-s1.pdf]

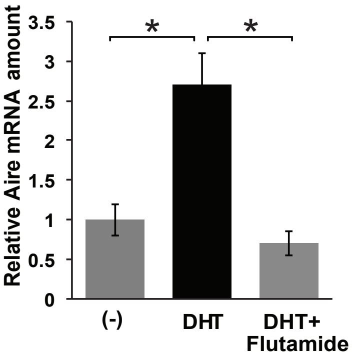

Supplementary Figure 1: Flutamide blocks DHT mediated increase in Aire expression. Relative Aire mRNA expression in primary mouse thymic stromal cells after culture with or without 10nM DHT or vehicle control (-) for 6 h. Cells were pretreated with or without 5 uM flutamide for 2 hours prior to DHT incubation. 10% charcoal-stripped serum was used in culture medium. Relative mRNA expression was measured by quantitative RT-PCR. *p<0.05.

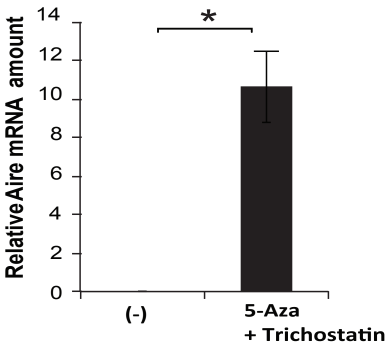

Supplementary Figure 2: 5 Aza and Trichostatin induce Aire transcription in LnCaP cells. Relative Aire mRNA expression in LNCaP cells treated for 24 hours with 5 mM 5-Aza, a DNA methyltransferase inhibitor, and 100 ng/mL trichostatin, a deacetylase inhibitor. Relative expression was measured by quantitative RT-PCR. *p<0.05.

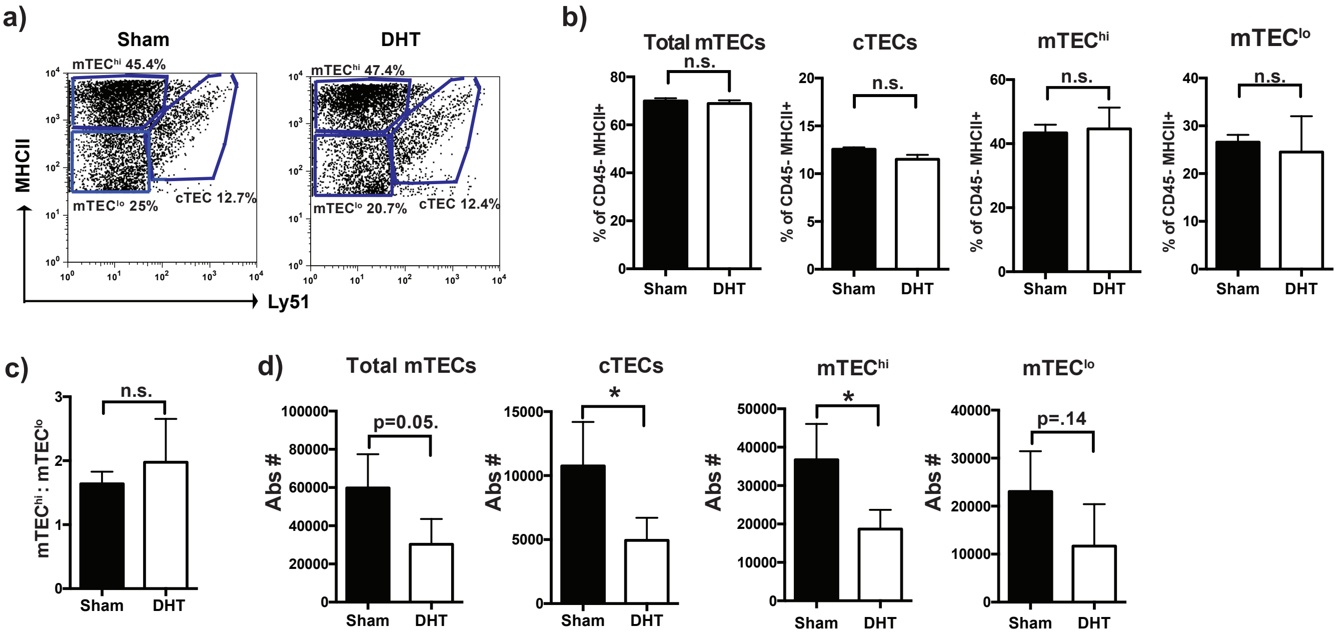

Supplementary Figure 3: DHT does not alter thymic epithelial subset frequencies, but decreases thymic epithelial numbers globally (a)Representative flow cytometric plots of MHCII and Ly51 expression in MHCII^{+} CD45^{-} thymic stromal cells of male mice subjected to sham treatment or DHT pellet insertion. Gating for mTEC^{hi}, mTEC^{lo}, and cTECs and corresponding frequencies are shown. (b) Average frequencies of total mTEC (mTEC^{hi} + mTEC^{lo}), cTEC, mTEC^{hi} cells, and mTEC^{lo} cells. (c) Average ratio of mTEC^{hi} : mTEC^{lo} cells. (d) Average absolute numbers of subsets in (b). n= 3-4 per group. n.s.= not significant. *p<0.05.

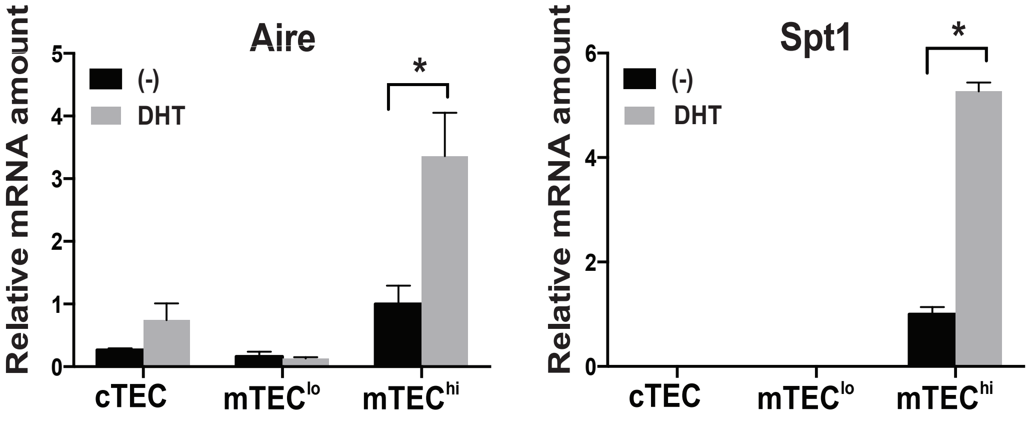

Supplementary Figure 4: DHT increases Aire and TSA expression in MHCII-high mTECs. (A and B) Relative Aire (A) and Spt1 (B) expression in sorted MHCII-high mTECs (mTEC^{hi}; CD45^{-} Ly51^{low}, MHCII^{high}), MHCII-low mTECs (mTEC^{lo}; CD45^{-} Ly51^{low}, MHCII^{low}), or cTEC (CD45^{-} Ly51^{high}, MHCII^{+}) cells by quantitative RT-PCR. Thymi are from mice treated with placebo (black bars) or 10 mg DHT pellet (gray bars). *$p<0.05$.

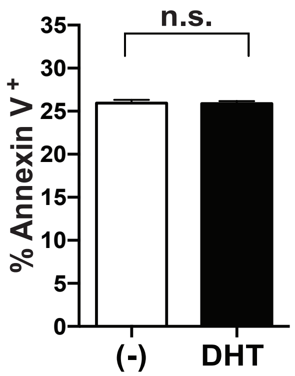

Supplementary Figure 5: **DHT is not directly toxic to thymocytes.** Thymocytes from Tyrp1^{B-w} TRP-1 TCR Tg Rag -/- mice, which are rescued from negative selection due to TRP-1 deficiency, were incubated with vehicle control (-) or 10nM DHT for six hours. The frequency of Annexin V^{+}thymocytes was determined by flow cytometry.

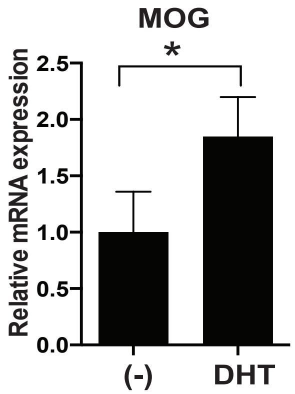

Supplementary Figure 6: DHT induces MOG expression in thymic stroma. Thymic stroma isolated from WT male C57BL/6 mice were incubated with either vehicle control (-) or 10nM DHT. Relative MOG mRNA expression was compared by real time RT-PCR.

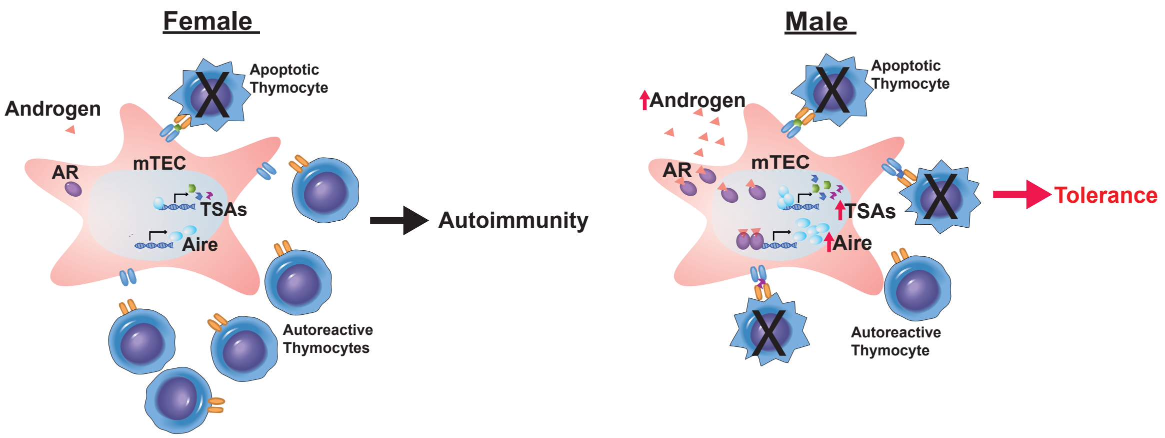

Supplementary Figure 7: Model for male protection from autoimmunity. A proposed model in which higher circulating androgen levels in males induce higher Aire and TSA expression in the thymus. More robust negative selection of self-reactive T cells protects males from autoimmune diseases.

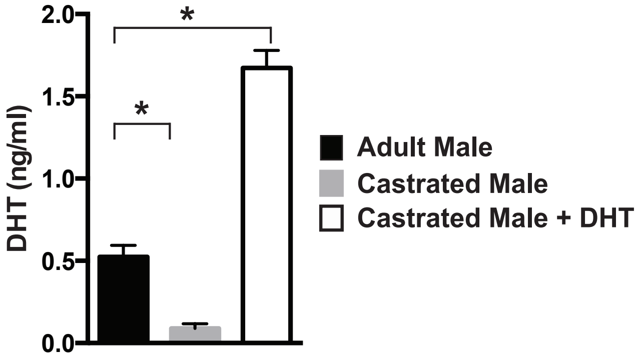

Supplementary Figure 8: Serum DHT levels. Circulating levels of DHT in unmanipulated adult mice, castrated male mice, and castrated male mice implanted with DHT pellet. *$p<0.05$.

Aire promoter	Mutations in Androgen Receptor Binding Sites								
	**-1032**	**-994**	**-970**	**-943**	**-391**	**-339**	**-260**	**-236**	**-52**
**WT**	GCACTGTCC	CTCCTGTCC	GGACACCTT	CTCCTGTCC	GGACAGGGC	TCTCTGTCC	GGACAGCAG	GAAGTGTCC	CCAGTGTCC
**Mut1**	GCACTGTCC	CTCCTGTCC	GGACACCTT	CTCCTGTCC	**C**GACA**A**GG**A**	TCTC**AAAA**C	**CGCGAA**CAG	GAAG**ACCAA**	CCAGTGTCC
**Mut2**	GCA**AAAA**C	CTCCT**AAA**C	**CGCA**ACCTT	CTCC**AAAA**C	GGACAGGGC	TCTCTGTCC	GGACAGCAG	GAAGTGTCC	CCAGTGTCC
**Mut3**	GCA**AAAA**C	CTCCT**AAA**C	**CGCA**ACCTT	CTCC**AAAA**C	**C**GACA**A**GG**A**	TCTC**AAAA**C	**CGCGAA**CAG	GAAG**ACCAA**	CCAGTGTCC
**Del**	- - - - - - - - - -	- - - - - - - - - -	- - - - - - - - - -	- - - - - - - - - -	- - - - - - - - - -	- - - - - - - - - -	- - - - - - - - - -	- - - - - - - - - -	- - - - - - - - - -

Supplementary Table 1: Sequences of wildtype and mutated AIRE 5' promoter regions in AIRE promoter-luciferase reporter constructs. Sequences at 9 potential AR binding sites (-1032, -994, -970, -943. -391, -339, -260, -236, and -52 relative to translational start site) in the AIRE 5' promoter region. Wildtype (WT) sequences for AR binding sites are shown at the top. For Mut1-3 constructs, multiple point mutations in the potential AR binding sites are shown in bold. The Del construct contains deletions (-) of all 9 AR binding sites.

	WT+Sham n=19	WT+DHT n=15	GW+Sham n=15	GW+DHT n=16
**Mean peak disease severity**	**3.2**	**2.4**	**3.7**	**3.7**
Experiment 1	3.0(0.25)<br>n=8	2.2(0.38)[a]<br>n=5	3.5(0.19)<br>n=5	3.4(0.19)<br>n=5
Experiment 2	3.6(0.2)<br>n=5	2.6(0.5)[a]<br>n=5	3.8(0.2)<br>n=5	4(0)<br>n=5
Experiment 3	3.8(0.4)<br>n=6	3(0.8)[a]<br>n=5	3.8(0.4)<br>n=5	3.3(1.0)<br>n=6
**Mean day of onset**	**8.8**	**10.8**	**8.6**	**9.4**
Experiment 1	9.6(0.3)	12.5(1.5)[a]	9.0(0.32)	9.2(0.2)
Experiment 2	8.4(0.2)	8.8(0.4)[a]	8.0(0)	8.6(0.3)
Experiment 3	8.5(0.5)	11(2.2)[a]	8.8(0.4)	10.6(1.3)
**Cumulative score***	**37.5**	**20.4**	**43.9**	**41.9**
Experiment 1	32.6(5.7)	15.8(5.3)[a]	42.0(4.0)	39.0(3.0)
Experiment 2	40.4(2.7)	25(4.0)[a]	45.8(1.4)	44.8(2.4)
Experiment 3	n/a	n/a	n/a	n/a
**Death from disease**	**1/19**	**0/15**	**1/15**	**2/10**
Experiment 1	0/8	0/5	0/5	0/5
Experiment 2	1/5	0/5	1/5	2/5
Experiment 3	0/6	0/5	0/5	0/6
**Incidence**	**19/19**	**14/15**	**15/15**	**16/16**
Experiment 1	8/8	4/5	5/5	5/5
Experiment 2	5/5	5/5	5/5	5/5
Experiment 3	6/6	5/5	5/5	6/6

Supplementary Table 2: MOG-induced EAE in male mice treated with DHT. Clinical EAE scores in DHT or sham treated WT or Aire-deficient $Aire^{GW/+}$ male mice. Values in bold are means or sums for three experiments. For each experiment, values are means (SE). [a]Significant difference from genotype-matched sham treated counterpart. *Sum of scores from Day 0 to 21. Cumulative scores are not available (n/a) for Experiment 3 because mice were sacrificed at Day 14.

	WT female n=15	WT male n=15	GW female n=15	GW male n=16
**Mean peak disease severity**	**3.3**	**2.3**	**3.3**	**3.2**
Experiment 1	3.6(0.25) n=5	2.4(0.25)[a] n=5	3.5(0.33) n=5	3.6(0.2) n=5
Experiment 2	2.8(0.5) n=5	1.8(0.25)[a] n=5	2.6(0.25) n=5	2.4(0.25) n=5
Experiment 3	3.5(0.33) n=5	2.6(0.5)[a] n=5	3.8(0.25) n=5	3.7(0.4) n=6
**Mean day of onset**	**14.5**	**17**	**13.4**	**13.5**
Experiment 1	12.8(0.6)	13.8(0.8)	11.2(0.8)	12.2(0.4)
Experiment 2	17.4(1.5)	20.8(1.9)	16.2(1.7)	16.2(1.5)
Experiment 3	13.3(0.8)	16.4(1.4)[a]	12.8(0.7)	12.1(1.1)
**Cumulative score**	**19.8**	**8.9**	**20.6**	**19.2**
Experiment 1	23.2(3.2)	13.6(2.2)[a]	28.3(4.2)	27.8(3.3)
Experiment 2	16.4(7.5)	4.2(2.2)[a]	12.8(4.0)	10.5(3.9)
Experiment 3	n/a	n/a	n/a	n/a
**Death from diease**	**0/15**	**0/15**	**1/15**	**1/15**
Experiment 1	0/5	0/5	0/5	0/5
Experiment 2	0/5	0/5	0/5	0/5
Experiment 3	0/5	0/5	1/5	1/5
**Incidence**	**15/15**	**15/15**	**15/15**	**15/15**
Experiment 1	5/5	5/5	5/5	5/5
Experiment 2	5/5	5/5	5/5	5/5
Experiment 3	5/5	5/5	5/5	5/5

Supplementary Table 3 : MOG-induced EAE in male and female mice. Clinical EAE scores in male and female WT and $Aire^{GW/+}$ mice. Values in bold are cumulative means or sums for three experiments. For each experiment, values are means (SE). [a]Significant difference from genotype-matched female counterpart. *Sum of scores from Day 0 to 21. Cumulative scores are not available (n/a) for Experiment 3 because mice were sacrificed at Day 14.

Region	Primer sequences	Orientation	Ref (PMID)
Aire promoter (-2916 to -2643)	TGGAGAGAAGGTCAGCAGGT	Forward	
	CCTCGTTGCAGTTGAGACAA	Reverse	
Aire promoter (-1130 to -867)	CTTTTCCGGAGCAATCTCAG	Forward	
	GTGGACGAAGGTACCAGGAA	Reverse	
Aire promoter (-383 to -164)	GCCACATTCGGAAGTGAGAG	Forward	
	GACCACGCAAAGAGCAAAG	Reverse	
Positive control 1: PSA enhancer	TGGGACAACTTGCAAACCTG	Forward	16137620
(-4212 to -4127)	CCAGAGTAGGTCTGTTTTCAATCCA	Reverse	
Positive control 2: PSA promotor	CCTAGATGAAGTCTCCATGAGCTACA	Forward	16137620
(-273 to -184)	GGGAGGGAGAGCTAGCACTTG	Reverse	
Negative control 1: -1575 to -1275	ACCTTCCTCCCCAGGTTTG	Forward	
	ATTAGGGCTGAAGGGTGGAC	Reverse	
Negative control 2: Aire exon 11	CTCGGGTTCGGGTTCAGCTA	Forward	
	CCCGCCGACCACGCTCACT	Reverse	

Supplementary Table 4: Primer sequences for ChIP.
